# Hypermethylation of Frizzled1 is associated with Wnt/β-catenin signaling inactivation in mesenchymal stem cells of patients with steroid-associated osteonecrosis

**DOI:** 10.1038/s12276-019-0220-8

**Published:** 2019-02-26

**Authors:** Fei Wu, Jing Jiao, Feng Liu, Yue Yang, Shanfeng Zhang, Zhenhua Fang, Zhipeng Dai, Zhibo Sun

**Affiliations:** 1 Department of Orthopedics, Renmin Hospital, Wuhan University, Wuhan, Hubei China; 2 Department of Orthopedics, Wuhan Fourth Hospital; Puai Hospital, Tongji Medical College, Huazhong University of Science and Technology, Wuhan, Hubei China; 3 Department of Orthopedics, Henan Provincial People’s Hospital, Zhengzhou, Henan China

**Keywords:** Stem-cell research, Calcium and phosphate metabolic disorders

## Abstract

The Wnt/β-catenin signaling pathway is associated with the pathogenesis of steroid-induced osteonecrosis. Our investigation studied whether aberrant CpG island hypermethylation of the FZD1 gene was present in patients with osteonecrosis of the femoral head (ONFH), which results in Wnt/β-catenin signaling inactivation and subsequent cell dysfunction. Bone marrow was collected from the proximal femurs of patients with steroid-associated ONFH (*n* = 21) and patients with new femoral neck fractures (*n* = 22), and then mesenchymal stem cells (MSCs) were isolated. We investigated cell viability, the transcription and translation levels of Wnt/β-catenin signaling-related genes, the extent of methylation at CpG islands of the FZD1 promoter, and the osteogenic and adipogenic differentiation abilities of MSCs from the control group and from the ONFH group treated with or without 5′-Aza-dC. According to the results, MSCs from the ONFH group showed a reduced proliferation ability, low transcription and translation levels of FZD1, inhibition of the Wnt/β-catenin signaling pathway, weakened osteogenesis and enhanced adipogenesis ability. Aberrant CpG island hypermethylation of FZD1 was observed in the ONFH group. Treatment with 5’-Aza-dC resulted in de novo FZD1 expression, reactivation of the Wnt/β-catenin signaling pathway and promotion of osteogenesis. Taken together, our study not only provides novel insights into the regulation of the Wnt/β-catenin signaling pathway in this disease but also reveals potential for the use of demethylating agents for the treatment of GC-associated ONFH.

## Bone disease: Signals from a modified gene

Studies of genetic and molecular signaling processes in the bone disease osteonecrosis, when it is associated with steroid use, reveal insights into disease development and suggest new approaches for treatment. Steroid drugs increase the risk of osteonecrosis, in which bone tissue dies due to insufficient blood supply, but the mechanism of this effect is unclear. Researchers in China, led by Zhibo Sun at Wuhan University, investigated a link between the aberrant addition of methyl groups (CH_3_) to the DNA of a specific gene and the onset of the disease. They identified an important molecular signaling pathway in cultured bone marrow cells from patients that is inhibited by the gene methylation. Treating these cells with a drug that inhibits methylation led to reactivation of the gene and the associated signalling pathway that promotes healthy bone formation.

## Introduction

Since glucocorticoids (GC) are widely used in daily clinical practice, their exogenous usage ranks first among the known risk factors for nontraumatic osteonecrosis of the femoral head, called glucocorticoid-induced osteonecrosis of femoral head (ONFH). Currently, several alternative mechanisms have been postulated for GC-induced ONFH, such as fat embolisation^1^, intramedullary pressure changes^2^, modified arterial constriction^3,4^, circulatory impairment^5^, coagulation disorders^6^, and cell dysfunction^7–9^. However, none of these can explain the underlying mechanism. Our previous study demonstrated that the abnormal differentiation of bone marrow mesenchymal stem cells (MSCs) is a principal mechanism involved in the onset and progression of this disease^10,11^.

MSCs, originally identified in adult bone marrow, can proliferate and differentiate into multiple mesodermal lineages, such as osteoblasts^12^, cardiocytes^13^,chondrocytes^14^, and adipocytes^15^. In GC-induced ONFH, decreased numbers, hypoproliferative activity and abnormal differentiation of MSCs of the femoral head, neck, and metaphysis result in poor self-repair and a negative prognosis. When the adipocyte population becomes more numerous than the osteoblast population, the intraosseous pressure may rise, ultimately causing ONFH. Therefore, to treat GC-associated ONFH, it is important to inhibit MSCs from undergoing adipogenesis and promote their osteogenesis.

The canonical Wnt signaling pathway plays a vital role in the regulation of bone homeostasis, which inhibits the differentiation of MSCs into chondrocytes and adipocytes while promoting osteoblastic differentiation^16–18^. This pathway controls both the proliferation and differentiation of osteoblastic precursors and maintains mature osteoblasts^19,20^. As a receptor for the Wnt signaling pathway, Frizzled1 (FZD1) plays an important role in osteoblast mineralization. Its promoter is regulated by several transcription factors, including Sp1, E2F1, and AP2^21–23^. Recent studies showed that FZD1 is closely related to bone mineral density (BMD)^24,25^. However, its expression in the MSCs of patients with steroid-induced ONFH has not been investigated yet.

In this study, we focused on FZD1 in MSCs and investigated whether FZD1-promoter hypermethylation is present in patients with ONFH and results in Wnt/β-catenin signaling inactivation and subsequent cell dysfunction. We performed methylation analysis of the promoter CpG islands of FZD1 by bisulfite sequencing (BSP) and investigated whether demethylation of FZD1 exerts a beneficial influence on cell viability and osteogenic differentiation. We attempted to confirm that FZD1-promoter hypermethylation is related to Wnt/β-catenin signaling inactivation in the development of GC-induced ONFH.

## Materials and Methods

### Patient recruitment

This study was approved by the Ethics Committee of Renmin Hospital. All methods were performed in accordance with the relevant guidelines and regulations of the authors’ institution. Between June 2016 and May 2017, 21 patients (10 men, 11 women; mean age 51.6, ranging from 38–63 years) with GC-induced ONFH were selected at the authors’ institution (Renmin Hospital, Wuhan, China), and 22 subjects with femoral neck fractures (11 men and 11 women; mean age 54.3, ranging from 37 to 70 years) were enrolled as controls. Clinical characteristics for all participants are summarized summarized in Table 1. For GC-induced ONFH, the steroid exposure threshold is 1800 mg GC or its equivalent over 4 weeks^26^. After written informed consent was obtained from patients, bone marrow aspirates (5 mL) were procured from the proximal ends of femurs while inserting a tapered awl into the femoral canal during hip replacement surgery.

**Table 1:** Clinical Characteristics of the subjects

Characteristics	ONFH (*n* = 21)	Controls (*n* = 22)	*P* value
Age (years)	51.6 ± 8.2	54.3 ± 8.3	0.278
Gender (male/female)	10/11	11/11	0.876
Body mass index (kg/m^2^)	25.9 ± 2.6	24.4 ± 2.7	0.057
Hypertension	3 (14.3%)	3 (13.6%)	0.951
Smoker	7 (33.3%)	8 (36.4%)	0.835
Alcoholism	3 (14.3%)	4 (13.6%)	0.729
Glucocorticoid medication	21 (100%)	2 (9%)	
Total cholesterol (mmol/L)	5.43 ± 0.54	4.82 ± 0.67	0.002
LDL cholesterol (mmol/L)	3.29 ± 0.38	3.22 ± 0.39	0.521
HDL cholesterol (mmol/L)	1.27 ± 0.37	1.38 ± 0.37	0.327
Triglycerides (mmol/L)	1.75 ± 0.40	1.60 ± 0.28	0.156

The data are mean ± standard derivation. Statistical significances of differences (*p* values) between groups were determined by paired Student’s *t* test (continuous values) and chi-square test (categorical values)

*GC* glucocorticoid, *LDL*, low-density lipoprotein, *HDL* high-density lipoprotein

### Cell culture

hMSCs were isolated from bone marrow aspirates and cultured as described previously^27^. Adherent cells were cultured for 12 to 14 days until they attained a confluence of greater than 80%. The cells were then digested with a solution of 0.25% trypsin and 0.02% EDTA (Invitrogen, Carlsbad, CA, USA) and replated at a 1:2 dilution for initial subculture. hMSCs underwent this treatment three times before they were collected for further use.

### Cell viability measurement

As described previously, MSCs were plated in 96-well plates at a density of 2 × 10^3^ cells per well^28^. After adherence to the plates, the initial defining medium was aspirated away and replaced with complete medium supplemented with 5’-Aza-dC (Sigma-Aldrich, St. Louis, MO) in the treatment group. At 24, 48, and 72 h, cell proliferation was assayed by MTT according to the manufacturer’s instructions.

### Immunofluorescent staining

Cells were fixed in 4% paraformaldehyde for 24 h, permeated with 0.2% Triton X-100 (Sigma), blocked, and then finally incubated with primary antibodies at a dilution of 1:100 at 4 °C overnight. Cells selected for DKK1, FZD1, and β-catenindetection were incubated with FITC- and Cy3-conjugated secondary antibodies at 37 °C for an additional one hour using standard concentrations from the supplier. Then, the slides were washed and mounted with CitiFluormountant (Agar Scientific, UK).

### Western blot

Cells were washed twice with ice-cold PBS, scraped into 0.2 mL of buffer (50 mM Tris with pH 7.4, 150 mM NaCl, 1% Triton X-100, 1% sodium deoxycholate, 0.1% SDS, and 0.05 mM EDTA), and incubated on ice for 20 min, followed by centrifugation at 12,000 rpm for 10 min. Protein concentrations were quantified by a BCA Protein Assay Kit (Beyotime Institute of Biotechnology, China). Afterwards, proteins were diluted to equal concentrations, boiled for 5 min, separated by 10% SDS-PAGE, and then blotted onto polyvinylidene fluoride (PVDF) membranes (Millipore, USA), which were probed with a FZD1 antibody (1:100, Abcam #ab71342), β-catenin antibody (1:1000, Abcam #ab16051), Runx2 antibody (1:1000, CST #12556) and PPARγ antibody (1:1000, CST #2435) overnight at 4 ℃. Membranes were incubated with horseradish peroxidase-conjugated secondary antibodies for 1 h at room temperature (Boster Biosciences, China). GAPDH was used to normalize for protein loading.

### Adipogenic and osteogenic differentiation

Adipogenic and osteogenic differentiation of human MSCs were performed as previously described^29^. For Oil red O staining, the cells were washed twice with PBS and fixed with 4% formaldehyde in PBS for 30 min at room temperature. Subsequently, they were stained for 1 h at room temperature with filtered Oil red O solution, washed twice with PBS, visualized under light microscopy and photographed. To extract the incorporated Oil red O, 1 mL of isopropanol was added to each well followed by 15 min of shaking at room temperature. After appropriate dilution, the absorbance of triplicate samples was read at 490 nm.

For ALP staining, the cells were washed twice with PBS and fixed with 4% formaldehyde in PBS for 30 min at room temperature. After three washes with PBS, ALP staining was performed by the addition of 5 ml of staining buffer (100 mM Tris-HCl, 150 mM NaCl, 1 mM MgCl_2_) containing chromogen substrate solution composed of 33 μl of 50 mg/ml nitro blue tetrazolium (NBT) and 16.5 μl of 50 mg/ml 5-bromo-4-chloro-3-indolyl phosphate (BCIP). Cells were stained with BCIP/NBT substrate for 30 min. Finally, the substrate solution was removed, and the cells were rinsed with deionized water, visualized under light microscopy and photographed.

For Alizarin Red S (ARS) staining, the cells were washed twice with PBS and fixed with 4% formaldehyde in PBS for 30 min at room temperature. After a brief wash with PBS, they were stained for 20 min with 40 mM ARS solution (pH 4.2). Next, they were rinsed five times with PBS to reduce nonspecific staining. Using Meta Morph imaging software (Universal Imaging, Downingtown, PA), osteogenic differentiation was quantified by measuring the area stained with Alizarin Red S. Measurements were performed in duplicate for each experiment, and experiments were repeated three times.

### Quantitative real-time PCR

Total RNA was extracted via standard protocols using standard commercial kits (TRIZOL® Reagent, Invitrogen, USA). Real-time PCR was performed using SYBR Green Master Mix according to the protocols of the supplier (Invitrogen, Carlsbad, CA, USA). The primer sequences are shown in Table 2. The SYBR Green signal was detected by a StepOne™ real-time PCR machine (ABI, USA). The relative levels of transcript expression were quantified using the ΔΔCt method. All real-time PCR was run in triplicate, and gene expression was analyzed using an ABI PRISM 7900HT Sequence Detection System (Applied Biosystems, USA).

**Table 2:** Primers used for real-time PCR

Genes	Sequence (5′→3′)	Product size
Real-time PCR		
*FZD1-F*	CTCGAGGTTTCCTCACTAGACAA	282 bp
*FZD1-R*	AATGGTTAAACCGCCCTAAATAA	
*Axin2-F*	GAGTGGACTTGTGCCGACTTCA	189 bp
*Axin2-R*	GGTGGCTGGTGCAAAGACATAG	
*Cyclin D1-F*	CCGTCCATGCGGAAGATC	86 bp
*Cyclin D1-R*	ATGGCCAGCGGGAAGAC	
*Runx2-F*	AGATGATGACACTGCCACCTCTG	125 bp
*Runx2-R*	GGGATGAAATGCTTGGGAACTGC	
*Osteocalcin-F*	CACTCCTCGCCCTATTGGC	112 bp
*Osteocalcin-R*	CCCTCCTGCTTGGACACAAAG	
*PPARγ-F*	CGAGAAGGAGAAGCTGTTGG	122 bp
*PPARγ-R*	TCAGCGGGAAGGACTTTATGTATG	
*GAPDH-F*	GGCACAGTCAAGGCTGAGAATG	143 bp
*GAPDH-R*	ATGGTGGTGAAGACGCCAGTA	

### Bisulfite sequencing

Bisulfite conversion was performed as previously described^30^. Briefly, total genomic DNA was isolated from MSCs using a DNeasy Tissue Kit (Cwbiotech). Two micrograms of genomic DNA were denatured in a volume of 50 μL by freshly prepared 0.3 M NaOH for 30 min at 42 ℃. After denaturation, 30 μL of freshly prepared hydroquinone (10 mM) and 510 μL of sodium bisulfite (3.6 M, pH 5.0) were added and incubated at 50 ℃ for 16 h. Modified DNA was purified using a DNeasy spin column (Qiagen) and eluted in a volume of 50 μL. This was followed by desulfonification by adding 5.5 μL of 3 M NaOH for 15 min at 37 ℃. Samples were neutralized by adding 33 μL of ammonium acetate (10 M, pH 7.0), followed by ethanol precipitation and resuspension in water.

PCR was performed at 95 ℃ for 5 min followed by 40 cycles of 95 ℃ for 30 s, 55 ℃ for 30 s and 72 ℃ for 1 min with a final extension at 72 ℃ for 7 min. Primer pairs were designed using a custom interface with primer3. The PCR products were tested in 2% agarose gel and then cloned into the pEASY-T1 vector (TransGen Biotech, Beijing, China). Colony PCR was undertaken to screen the positive colonies. Clones with correctly sized PCR products were sequenced on an ABI sequencer with dye terminators (Applied Biosystems, Foster City, CA, USA). Based on the sequencing results for ten clones, the methylation frequency was determined for each CpG site.

### Statistical analysis

Statistical analysis was carried out via SPSS version 13.0 for Windows (SPSS, Chicago, IL, USA). Significant differences were determined using either the Mann-Whitney U test or the Wilcoxon signed-rank test. Data are presented as the mean ± standard deviation. Probabilities lower than 5% (*P* < 0.05) were considered statistically significant. All experiments were repeated three or more times.

## Results

### Measurement of hMSC viability

**Fig. 1:**
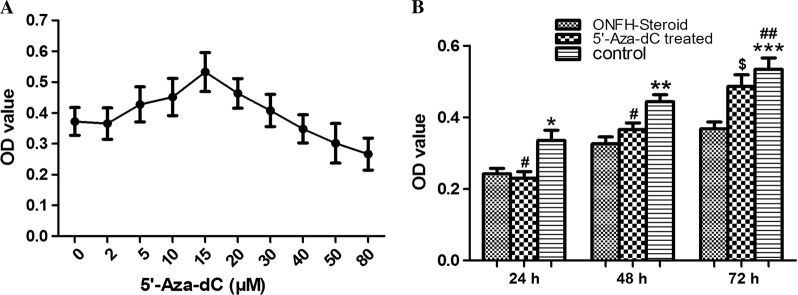
Dose response curves for 5’-Aza-dC-treated cultures and a cell proliferation assay. **a** Cellular viability reached its peak at the dosage of 15 μM at *t* = 72 h. **b** The proliferation capacity of MSCs at *t* = 24 h, 48 h and 72 h. *P < 0.05 vs. ONFH group, 5’-Aza-dC group at *t* = 24 h; **P < 0.01 vs. ONFH group at *t* = 48 h; ***P < 0.001 vs. ONFH group at *t* = 72 h; ^#^*P* > 0.05 vs. ONFH group at *t* = 24 h, 48 h; ^##^*P* > 0.05 vs. 5’-Aza-dC group at *t* = 72 h; ^$^*P* < 0.01 vs. ONFH group at *t* = 72 h

According to Fig. 1a, cellular viability reached its peak at the dosage of 15 μM, and obvious decreases were seen at dosages of 30 μM and above. Hence, the concentration of 15 μM was considered moderate and chosen for use in subsequent experiments.

As shown in Fig. 1b, the cellular viability in the control group was remarkably stronger than that in the GC-induced ONFH group at three different time points (*P* < 0.05). Following treatment with 15 μM 5’-Aza-dC, the cellular viability in the GC-induced ONFH group increased by more than one fourth but was still a little lower than that in the control group at t = 72 h (*P* < 0.05).

### Immunofluorescent staining

**Fig. 2:**
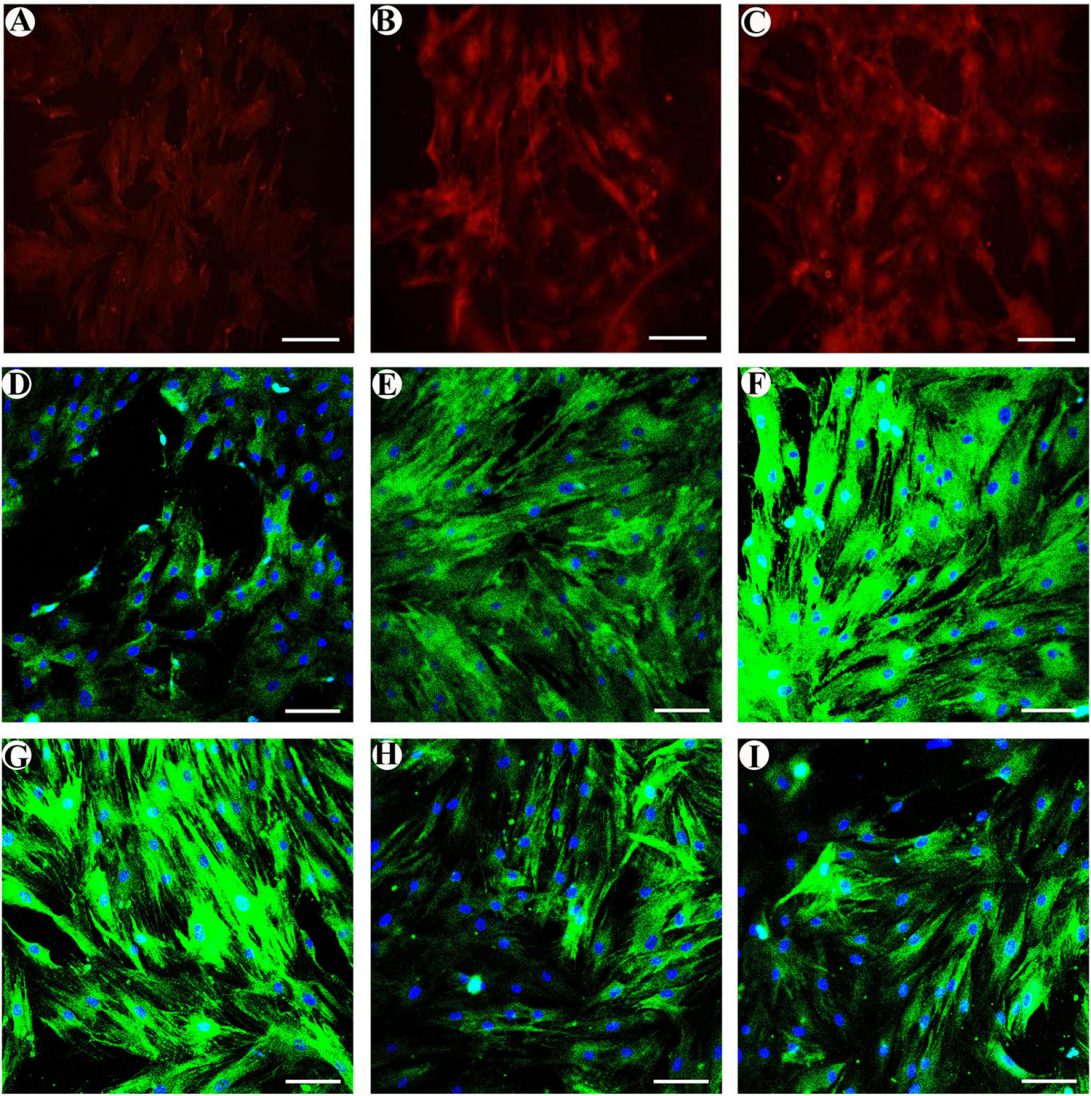
Immunofluorescent staining. MSCs were stained with β-catenin (red), FZD1 (green) and DKK1 (green) antibodies (green), and the cells were co-stained with DAPI to indicate the nuclei (blue). **a**–**c** β-catenin showed obviously weak staining in the ONFH group. Following treatment with 5′-Aza-dC, β-catenin staining was obviously increased but was still weaker than that of the control group. **d**–**f** FZD1 showed obviously weak staining in the ONFH group. Following treatment with 5’-Aza-dC, FZD1 staining was obviously increased but was also weaker than that of the control group. **g**–**i** Dkk1 had strong staining in the ONFH group. Treatment with 5′-Aza-dC obviously decreased Dkk1 staining. The control group had weak Dkk1 staining (*bars* 50 μm)

Compared to the control group, FZD1 and β-catenin showed obviously weak staining and Dkk1showed strong staining in the ONFH group. Following treatment with 5’-Aza-dC, β-catenin and FZD1 staining was obviously increased but was still weaker than that of the control group (Fig. 2a–f). However, 5′-Aza-dC treatment obviously decreased Dkk1 staining. The control group had weak Dkk1 staining (Fig. 2g–i). In addition, compared to the ONFH group, we found that 5’-Aza-dC increased the accumulation of β-catenin-Cy3 complexes in the nucleus, but this accumulation was still lower than that of the control group.

### Adipogenic and osteogenic differentiation

**Fig. 3:**
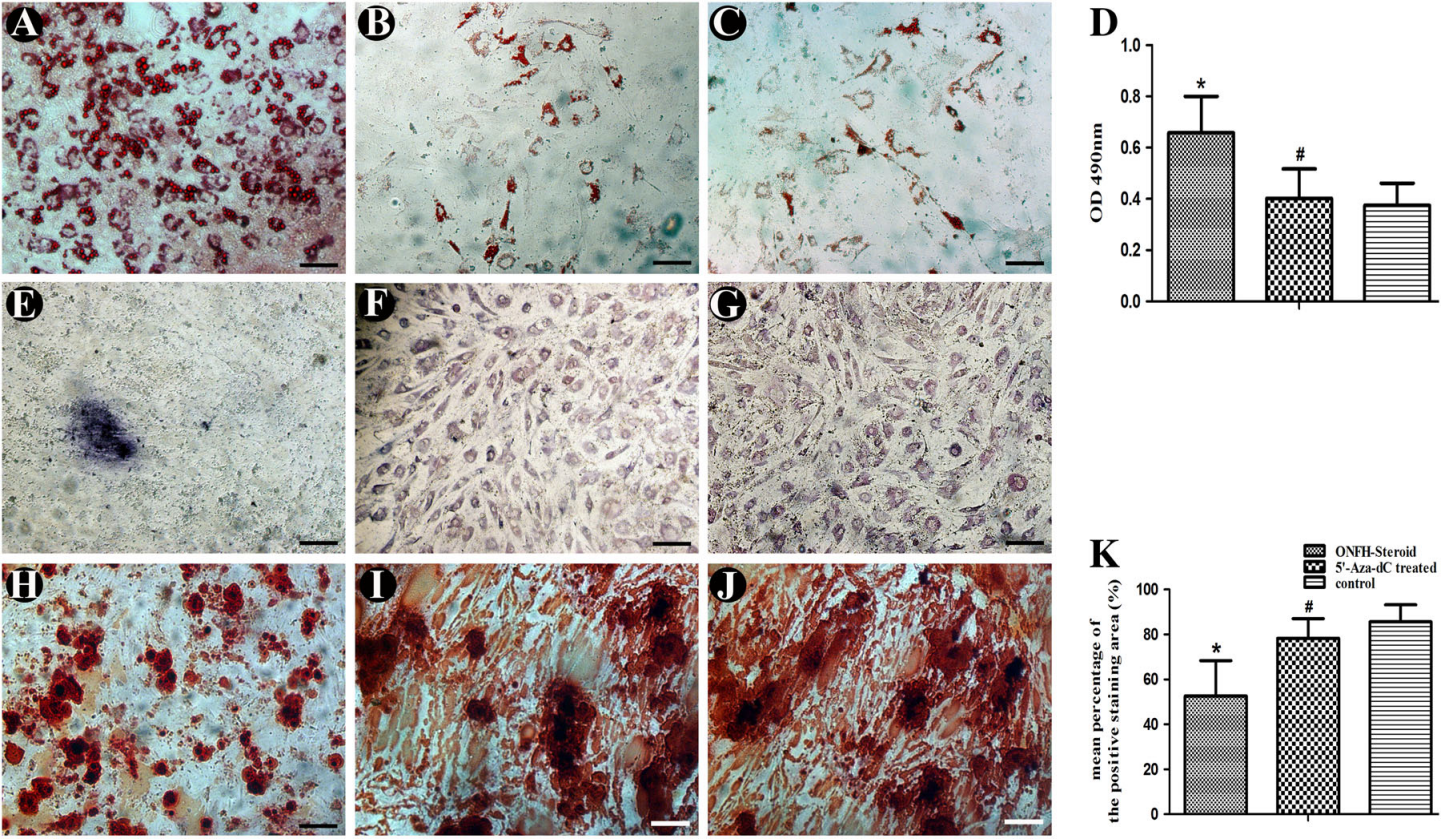
Adipogenic and osteogenic differentiation. MSCs from all groups were induced to adipocytes and osteoblasts for 21 days. **a** Oil red O staining of the ONFH group. **b** Oil red O staining of the 5’-Aza-dC group. **c** Oil red O staining of the control group. **d** Incorporated Oil red O was extracted with isopropanol, and quantification was performed as described in the “Methods” section. **e** BCIP/NBT staining of the ONFH group. **f** BCIP/NBT staining of the 5′-Aza-dC group. **g** BCIP/NBT staining of the control group. **h** Alizarin red S staining of the ONFH group. (**i**) Alizarin red S staining of the 5’-Aza-dC group. **j** Alizarin red S staining of the control group. **k** The area stained with alizarin red S was measured as described in the Methods. Data are represented as the mean value ± standard deviation. *P < 0.01 vs. 5’-Aza-dC group, control group. ^#^*P* < 0.05 vs. control group (*bars* 100 μm)

As shown in Fig. 3a–d, compared to control group, oil red O showed strong deposition in the GC-associated ONFH group after induction for 3weeks (*P* < 0.05). Treatment with 15 μM 5’-Aza-dC resulted in weakened staining, which was almost the same level as that of the control group (*P* > 0.05).

As shown in Fig. 3e–g, widespread blue-purple staining could be detected in the 5’-Aza-dC and control groups with light microscopy after conversion of the substrate BCIP/NBT by ALP. However, positive staining was hardly observed in the ONFH group.

As shown in Fig. 3h–k, ARS staining of the GC-associated ONFH group was weakest among all the groups. Treatment with 5’-Aza-dC resulted in alizarin red S staining with deeper intensity, which indicated enhanced calcium mineralization compared with that of the GC-associated ONFH group (*P* < 0.05).

### Western blot

**Fig. 4:**
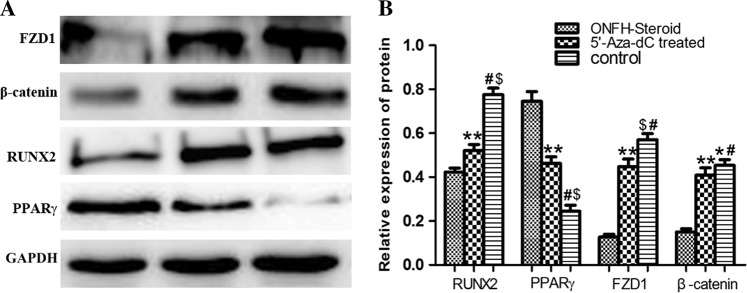
Western blot. **a** Western blot analysis of FZD1, β-catenin, Runx2 and PPARγ. **b** Gray level analysis of western blot. *P < 0.05 vs. 5’-Aza-dC group. ^#^*P* < 0.001 vs. ONFH group.^$^*P* < 0.01 vs. 5′-Aza-dC group, 5′-Aza-dC group. **P < 0.001 vs. ONFH group

Our results showed that 5’-Aza-dC markedly promoted FZD1, β-catenin and Runx2 translation while inhibiting PPARγ translation. It was observed that MSCs in the ONFH group showed obviously low expression of FZD1 and β-catenin compared to that of the control group. When treated with 5’-Aza-dC for 72 h, this expression correspondingly increased (Fig. 4). Cells in the ONFH group had strong PPARγ expression and weak Runx2 expression compared to that of the control group. Following pretreatment with 5’-Aza-dC for 72 h, this expression reached nearly the same levels as those of the control group.

### Quantitative real-time PCR

**Fig. 5:**
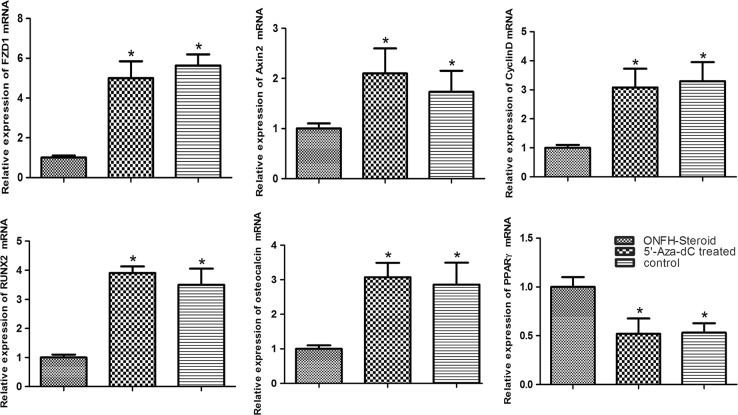
Real-time PCR. The transcript levels of osteogenesis, adipogenesis and Wnt/β-catenin signaling pathway-related genes in the three groups. Data are represented as the mean value ± standard deviation. *P < 0.01 vs. ONFH group

Our results showed that 15 μM5’-Aza-dC markedly promoted the transcription of FZD1, CyclinD1, Axin2, and osteogenesis-related genes (RUNX2, osteocalcin) while inhibiting adipogenesis-related gene (PPARγ) transcription. As shown in Fig. 5, the amount of FZD1 transcripts in the ONFH group was the lowest among all groups (*P* < 0.01), reaching a level nearly 5-fold higher at 72 h after treatment with 5’-Aza-dC, which was almost the same level as that of the control group (*P* > 0.05). Meantime, CyclinD1 and Axin2 transcription was elevated to varying degrees by treatment with 5’-Aza-dC. Pretreatment with 5’-Aza-dC also helped to promote RUNX2 and osteocalcin transcription and decrease PPARγ transcription.

### Bisulfite sequencing

**Fig. 6:**
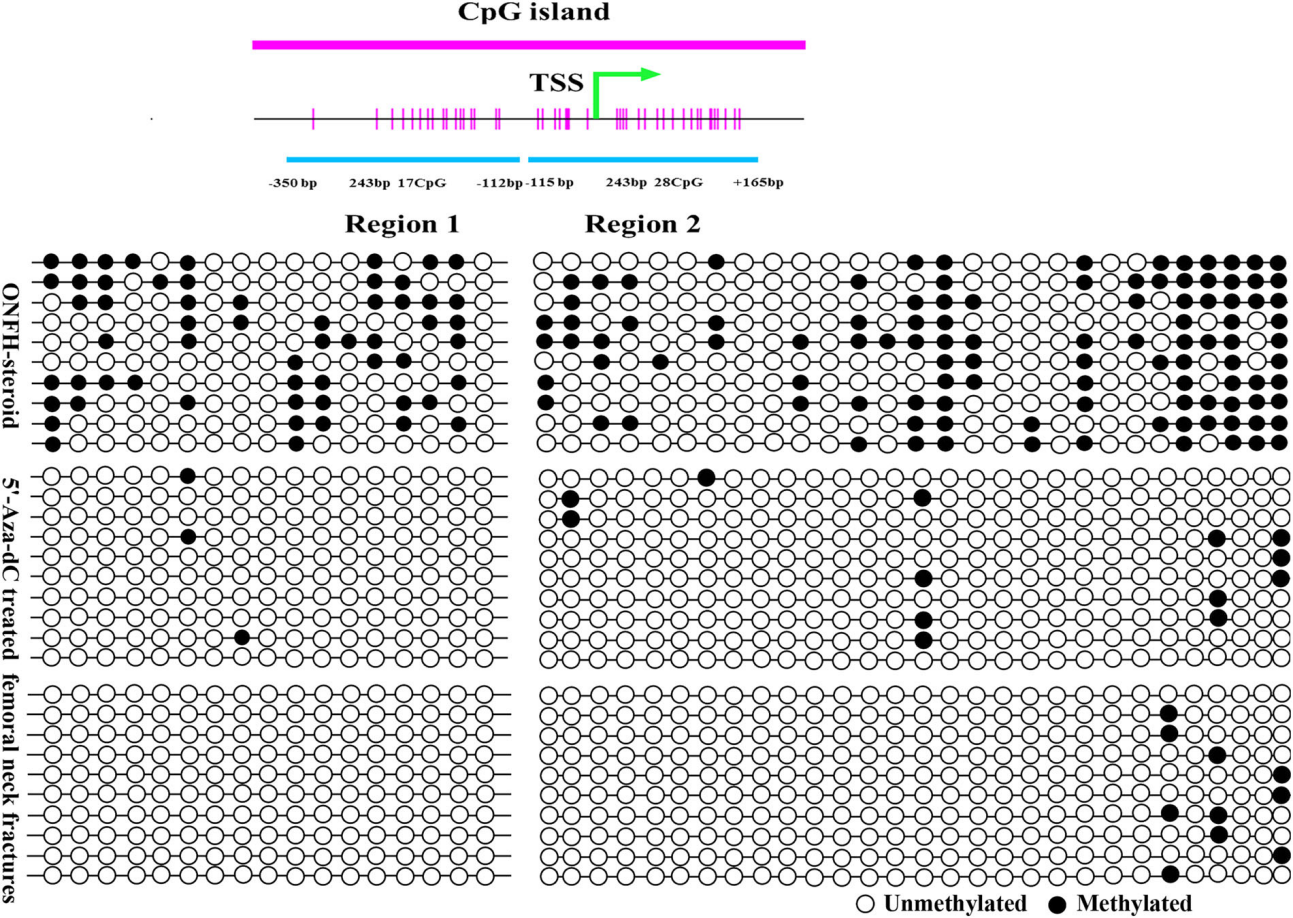
Bisulfite sequencing. Region 1 (−350 to −112 bp): The methylation ratios were 33.0% in the ONFH group, 1.8% in the 5’-Aza-dC group and 0 in the control group. Region 2 (−115 to + 165 bp): The methylation ratios were 38.6% in the ONFH group, 4.6% in the 5’-Aza-dC group and 3.6% in the control group

To determine whether the increased expression of FZD1 in MSCs had an underlying epigenetic basis, DNA methylation status in the FZD1 promoter was examined by bisulfite sequencing. A schematic overview of the promoter structure is shown in Fig. 6. Two regions in the promoter were selected: region 1, -350 to -112, and region 2, -115 to + 165. CpG hypermethylation was detected within the CpG island in the GC-associated ONFH group. After treatment with 15 μM 5′-Aza-dC for 72 h, the methylation ratio of region 1 decreased from 33.0 to 1.8%, while the methylation level of region 2 was clearly reduced from 38.6 to 4.6%. The methylation ratios of the control group were 0 (region 1) and 3.6% (region 2) (Fig. 6).

## Discussion

Currently, we see an increased incidence of ONFH due to the widespread use of exogenous glucocorticoids. At the present time, the precise mechanism underlying steroid-associated ONFH has not yet been elucidated, although numerous studies have been done. Epigenetics, an effective method to study the interplay between environmental signals and the genome, has received a great deal of recent attention. Our previous studies demonstrated that DNA hypermethylation plays an important role in the pathophysiology of GC-associated ONFH. It is beneficial for dysfunctional MSCs to reverse DNA hypermethylation modifications^10,11^. However, the exact mechanism has not yet been clarified.

The Wnt/β-catenin signaling pathway is reported to be involved in the pathogenesis of early-stage ONFH^31^. Mutations in components of the Wnt/β-catenin pathway are associated with decreased bone mineral density and increased fracture incidence as well as other skeletal disorders^32^. Dickkopf-1 (DKK1), one of the Wnt inhibitors, showed increased expression in ONFH patients with histories of corticosteroid and alcohol intake^33^. Some studies demonstrated that FZD1 is very important in osteoblast differentiation and mineralization, and its promoter can be regulated by several transcription factors^21–23^. Our pilot study showed that FZD1 was obviously decreased at the transcription and translation levels in GC-associated ONFH. Therefore, we hypothesized that FZD1 promoter hypermethylation resulted in low expression of FZD1, downregulation of the Wnt/β-catenin signaling pathway and MSC dysfunction in the MSCs of patients with GC-associated ONFH. Reversal of the hypermethylation status of the FZD1 promoter is helpful for reactivation of the Wnt/β-catenin signaling pathway and improvement of MSC osteogenic differentiation.

In this study, we assessed methylation at CpG islands of the *FZD1* gene in MSCs from 43 patients with GC-induced ONFH and femoral neck fractures. We found that the methylation ratio of the FZD1 promoter in the GC-induced ONFH group was obviously higher than that in femoral neck fractures group. Additionally, FZD1 mRNA and protein levels in the GC-induced ONFH group were one sixth and one tenth of those in femoral neck fractures group, respectively. Apparently, expression of the FZD1 gene was partly inhibited due to the promoter hypermethylation in the GC-induced ONFH group.

5′-Aza-dC, an inhibitor of DNA methyltransferase (DNMT), was demonstrated to be involved in cell proliferation and differentiation^34^. DNA methylation is a key mechanism associated with pluripotency and self-renewal of stem cells. It was reported that 5-Aza induced the direct conversion of adult gingival MSCs into cells of three embryonic lineages, suggesting their potential use for autologous cell therapy^35^. Cho et al. demonstrated that epigenetic modification induced by 5’-Aza-dC permitted direct programming of adipocytes into osteoblasts in a mouse model of osteoporosis, indicating that 5’-Aza-dC is a promising treatment for osteoporosis^36^.

Notably, we observed that cellular viability in the GC-induced ONFH group was remarkably lower than that in the control group at three different time points. Following treatment with 15 μM 5′-Aza-dC, cellular viability in the ONFH group increased by more than one fourth, while methylation ratios decreased to 1.8% (region 1) and 4.6% (region 2). This finding is consistent with our previous article^10,11^ and the recent report from Katarzyna Kornicka et al.^37^, who found that 5-azacytidine reduced reactive oxygen species accumulation, ameliorated superoxide dismutase activity, increased cellular proliferation and the BCL-2/BAX ratio, and ultimately slowed down and even reversed aged-related degenerative changes in MSCs. Our previous data showed the similar effects of 5-azacytidine on the MSCs of patients with GC-associated ONFH.

From an immunofluorescence analysis, we found that the MSCs of patients with GC-associated ONFH demonstrated hampered intranuclear translocation of β-catenin compared to that of the control group. 5’-Aza-dC can promote the intranuclear translocation of β-catenin. FZD1 was weakly expressed in the MSCs of patients with GC-associated ONFH, and this expression was increased by treatment with 5’-Aza-dC. However, expression of the DKK1 protein exhibited an opposite pattern. The PCR results showed that 5’-Aza-dC also increased the mRNA expression of FZD1, Axin 2, Cyclin D1 and Runx2, which were decreased in GC-associated ONFH. Moreover, MSCs treated with 5’-Aza-dC showed weakened adipogenesis and enhanced osteogenesis according to the oil red O, ALP and ARS staining results. Taken together, there is a good possibility that the beneficial effects of 5’-Aza-dC on GC-associated ONFH are attributable to FZD1-promoter demethylation of MSCs and reactivation of the Wnt/β-catenin signaling pathway. However, the underlying mechanism must be studied further.

In summary, this study has demonstrated that aberrant CpG island hypermethylation of the *FZD1* gene is present in patients with ONFH, resulting in Wnt/β-catenin signaling inactivation and subsequent cell dysfunction. By comparative observation, we confirmed that 5’-Aza-dC, at a suitable concentration, benefits the MSCs of patients with GC-associated ONFH by inducing de novo FZD1 expression. This study has provided new information regarding treatment targeting epigenetic changes in ONFH. However, more specific in vivo studies are needed to further elucidate the molecular pathomechanism underlying the development of ONFH.
